# Quantification of Ethylene Production in Leaf and Bud Tissue of the Subtropical Tree Crop Litchi (*Litchi chinensis* Sonn.) Using Gas Chromatography and Flame Ionization Detection

**DOI:** 10.21769/BioProtoc.4636

**Published:** 2023-03-20

**Authors:** Regina B. Cronje, Arnoldus J. Jonker

**Affiliations:** Agricultural Research Council – Tropical and Subtropical Crops, Mbombela, South Africa

**Keywords:** Ethylene, Gas chromatography, Flame ionization detector, Headspace, Leaves, Apical buds, Subtropical crops

## Abstract

Ethylene is an important plant hormone that is involved in the regulation of numerous processes in plant development. It also acts as a signaling molecule in response to biotic and abiotic stress conditions. Most studies have investigated ethylene evolution of harvested fruit or small herbaceous plants under controlled conditions, but only a few explored ethylene release in other plant tissues, such as leaves and buds, particularly those of subtropical crops. However, in light of increasing environmental challenges in agriculture (such as temperature extremes, droughts, floods, and high solar radiation), studies on these challenges and on potential chemical treatments for mitigating their effects on plant physiology have become more and more important. Thus, adequate techniques for the sampling and analysis of tree crops are needed to ensure accurate ethylene quantification. As part of a study on ethephon as a mitigating agent to improve litchi flowering under warm winter conditions, a protocol was developed for ethylene quantification in leaf and bud tissue of litchi following ethephon application, taking into account that these plant organs release lower ethylene concentrations than fruit. At sampling, leaves and buds were placed in glass vials of appropriate sizes for the respective plant tissue volumes and allowed to equilibrate for 10 min to release possible wound ethylene before incubating the samples for 3 h at ambient temperature. Thereafter, ethylene samples were aspirated from the vials and analyzed using a gas chromatograph with flame ionization detection, the TG-BOND Q+ column for separation of ethylene, and helium as the carrier gas. Quantification was achieved based on a standard curve derived from an external standard gas calibration with certified ethylene gas. This protocol will also be appropriate for other tree crops with similar plant materials as study foci. It will enable researchers to accurately determine ethylene production in various studies investigating the role of ethylene in general plant physiology or stress-induced plant responses following a range of treatment conditions.

## Background

Ethylene is a gaseous plant hormone that plays an important role in the regulation of numerous physiological and developmental plant processes, such as seed germination, root and shoot development, cellular growth regulation, carbon assimilation, senescence of leaves and flowers, organ abscission, and fruit ripening (Abeles et al., 1992; Pierik et al., 2006; Olsen, 2010; Wang et al., 2013; Dubois et al., 2018). Ethylene also acts as a signaling molecule in responses to biotic and abiotic environmental stresses, such as temperature extremes, droughts, floods, shading, radiation, nutrient deficiency, and mechanical and chemical damage (Yang and Hoffman, 1984; Iqbal et al., 2013; Dubois et al., 2018). The amount of ethylene production depends on the plant species, developmental stage of the plant, and organ type (Cristescu et al., 2013). Apart from endogenous or stress-induced ethylene production, production in plant organs can also be induced by exogenous application of the plant growth regulator ethephon (2-chloroethylphosphonic acid). Ethephon has been widely used in agriculture for different applications, including fruit ripening and coloration, organ abscission to aid harvesting, bloom delay, and suppression of vegetative growth (Nickell, 1994).

Accurate quantification of ethylene evolution from ethephon as well as endogenous ethylene production following different treatments is necessary to correlate production with respective plant responses and elucidate the functions and role of ethylene. According to Cristescu et al. (2013), there are three main methods to detect ethylene in plants: gas chromatography detection, electrochemical detection, and optical detection. These can be used for periodic or continuous measurement depending on the type of plant material and experimental design. Gas chromatography (GC) is most widely used for separation and analysis of ethylene, due to its small sample requirement, high selectivity, and fast analysis. Although more sensitive detection techniques have been developed, such as a laser-based sensing technique (Cristescu et al., 2013; Gwanpua et al., 2018), GC detection is still the most applicable method for tree crops, since non-destructive and/or continuous sampling is not possible. This applies particularly to studies that focus on trees in orchard systems and that are subject to climatic effects.

In fruit tree crops, the quantification of ethylene has mainly been used to assess the effect of endogenous ethylene production in developing fruit, e.g., on fruit coloration (Yin et al., 2001; Chervin et al., 2005; Wang et al., 2007), or in harvested fruit, e.g., on fruit quality and shelf life after harvest (Tseng et al., 2000; Gwanpua et al., 2018). However, few studies investigated ethylene production in leaves, buds, flowers, or other vegetative material from tree crops. Examples are GC detection of ethylene in leaves of citrus (Tudela and Primo-Millo, 1992), in shoot portions of apple (Sanyal and Bangerth, 1998), and in floral buds and leaves of mango (Bindu et al., 2017), peach (Liu et al., 2021) and litchi (Cronje et al., 2022) trees. However, most of these studies only provided limited information on the sampling and analysis techniques used to successfully reproduce the techniques for application in other crops with similar plant organs. For this purpose, we developed a protocol to easily determine ethylene evolution in leaves and apical buds of litchi as part of a recent study, which used ethephon to induce bud dormancy and delay panicle emergence for more consistent floral initiation in litchi (Cronje et al., 2022). To determine the mode-of-action of ethylene inside the leaf and bud tissue, as well as the downstream processes, such as relative expression of ethylene-, dormancy-, and flowering-related genes, ethylene concentration needed to be quantified reliably and accurately in these plant organs to correlate the results with those obtained from corresponding biochemical and molecular analyzes (Cronje et al., 2022). The sampling technique and incubation period was adapted specifically for litchi to account for low overall ethylene production and wound ethylene release after detaching of the respective plant organs. The quantification protocol was derived from a protocol for the quantification of ethylene evolution in tomato leaves developed by Kim et al. (2016) and used a porous layer open tubular (PLOT) TG-Bond Q+ column (Thermo Scientific) for direct separation of ethylene. While several related studies used packed alumina (Al_2_O_3_) columns that are recognized for the separation of hydrocarbons from C1 to C5, the use of the mentioned PLOT column is considered advantageous since it is specifically developed for selective separation of acetylene (C_2_H_2_), ethylene (C_2_H_4_) and ethane (C_2_H_6_) to baseline. Additionally, the use of a capillary column in conjunction with a flame ionization detector has proven to be highly sensitive with a linear calibration range of three orders of magnitude, i.e., 0.08 nL to 80 nL of injected C_2_H_4_. Although the protocol was specifically developed for leaves and buds of litchi, it can be equally applied to plant material from other fruit tree crops by making appropriate changes to container sizes.

## Materials and Reagents

1. 100 μL gas tight syringe with needle: 50 mm length, 23 gauge, point style 5, side hole (Thermo Scientific, catalog number: 36520050)
2. 2,500 μL gas tight syringe with needle: 65 mm length, 23 gauge, point style 5, side hole (Thermo Scientific, catalog number: 365Q2131)
3. 20 mL crimp top headspace vials (Thermo Scientific, catalog number: CHCV20-14)
4. 20 mm Si/PTFE septa (Cronus, catalog number: VCS-2004-1000)
5. 20 mm Al crimp cap (Cronus, catalog number: VCC-2002CS-1000)
6. 20 mm magnetic crimp cap (Cronus, catalog number: VCC-2002BM-500)
7. 20 mm hand crimper (Cronus, catalog number: VTC-20)
8. 1.8 mL, 9 mm screw top vials (Cronus, catalog number: VZS-0209C-100)
9. 9 mm PTFE/red rubber screw thread closure (Cronus, catalog number VKB-0203-09CB-5000)
10. 51 mm, 23 gauge Hamilton needles, point style 5, side hole (Hamilton, catalog number: 7729-06)
11. 17 mm injection port septum (Thermo Scientific, catalog number: 31303211)
12. 3 ml disposable plastic syringes (generic)
13. Plastic 2-way valves with luer locks and needle adaptor for syringes (Vernier, catalog number: PS-2WAY)
14. High purity helium (He) gas, 99.999% (Afrox, Baseline 5.0, catalog number: 524203-SE-C)
15. 79 μL·L^-1^ C_2_H_4_ gas standard, balance nitrogen (N_2_) gas (Afrox, catalog number: GOC mix 3292)
16. TG-BOND Q+ porous layer open tubular capillary column (Thermo Scientific, catalog number: 26005-6030)
17. 0.32 mm graphite encapsulated ferrules (Thermo Scientific, catalog number: 29053487)
18. 5 mm glass split liner (Thermo Scientific, catalog number: 45350030)
19. Graphite liner seal (Thermo Scientific, catalog number: 29033406)

## Equipment

1. Long nose pliers (generic)
2. Straight Iris scissors (generic)
3. Gas chromatograph with a flame ionization detector (Thermo Scientific, model: Trace GC Ultra)
4. Autosampler (Thermo Scientific, model: Triplus RSH)
5. Headspace tool (Thermo Scientific, catalog number: 1R77010-1125)
6. Air generator (Peak Scientific, model: Precision Zero Air)
7. N_2_ generator (Peak Scientific, model: Precision Nitrogen)
8. H_2_ generator (Peak Scientific, model: Precision Hydrogen)
9. 2-stage gas regulator with gauges and inlet stem with 5/8’’ left hand BSP nut (Afrox, catalog number: W019220)
10. Analytical balance (Adam Precision, model: PW184)

## Software

1. Thermo Xcaliber 3.0.63.3 (Thermo Fisher Scientific Inc.)
2. Excel (Microsoft Office 2016)

## Procedure

- **Hardware setup of gas chromatograph (GC)**
  1. Follow the manufacturer’s instruction manual to perform the following setup of the GC:
    - Install a 5 mm glass split liner together with a graphite seal into the split/splitless injector.
    - Place a 17 mm coated septum between the septum support and septum holder and hand tighten the injector cap. A diagram together with a photograph of the layout and components that form part of the injector is presented in Figure 1.
      **Figure 1.**
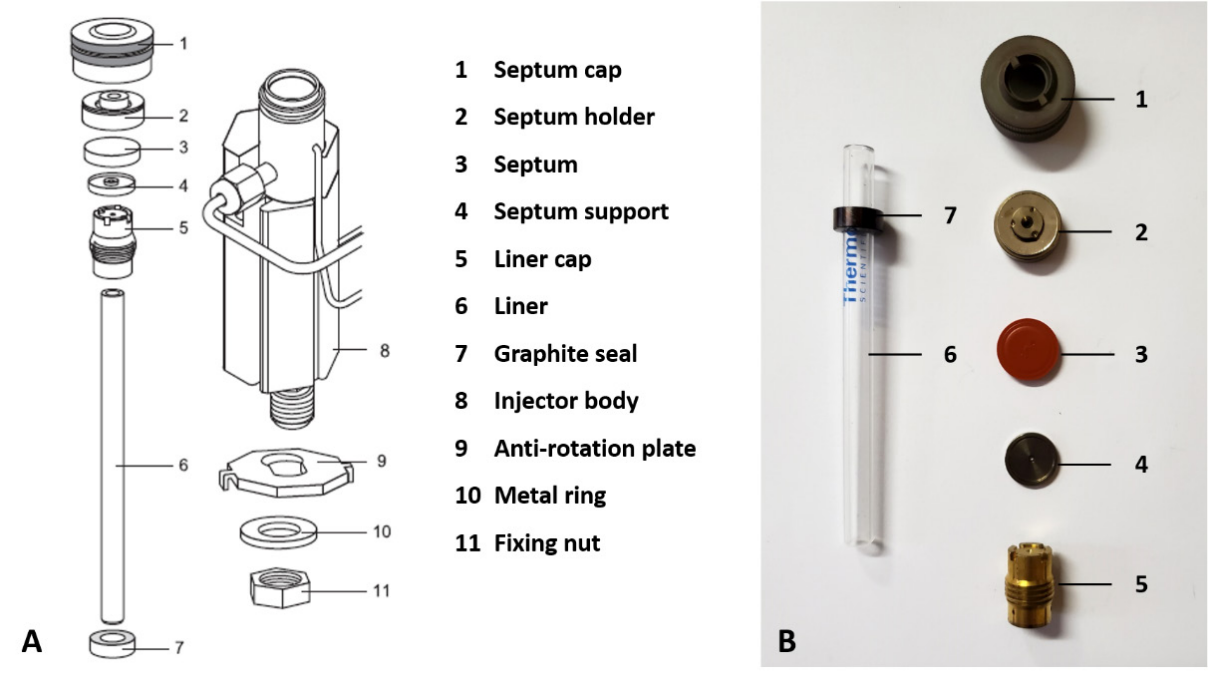
Components and layout of the GC’s split/splitless injector. A. Diagram reprinted from Thermo Scientific (2010). B. Photograph of components.
    - Connect the TG-BOND Q+ capillary column between the flame ionization detector (FID) and split liner using 0.32 mm graphite encapsulated ferrules. The column insertion depths are 40 mm and 94 mm into the split liner and FID, respectively. Figure 2 illustrates the installed column inside the oven, as well as how to determine the correct column insertion depths into the injector and FID.
      **Figure 2.**
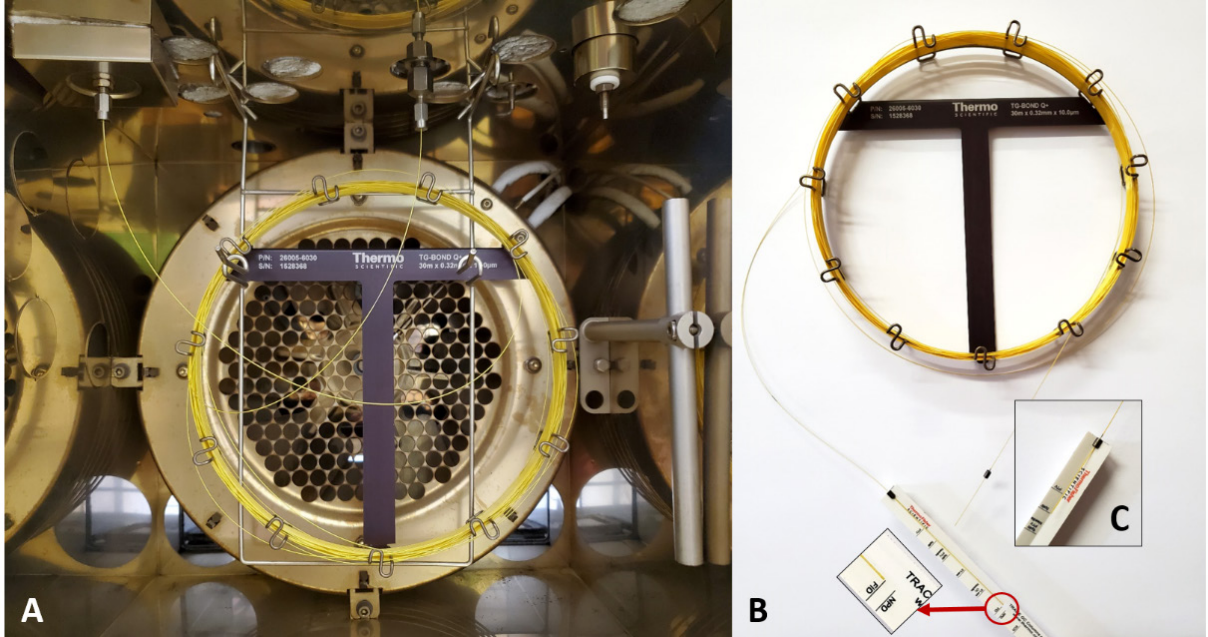
Installation of the TG-BOND Q+ capillary column. A. View of the column as installed in the GC’s oven between the split/splitless injector and the flame ionization detector (FID). B. Measuring the insertion depth of the column for the FID. C. Details of the position of the ferrule when gauging the column insertion depth for the injector.
    - Confirm that a supply of high purity He gas at a pressure of 800 kPa is connected to the GC.
    - Check operation of the Peak Scientific gas generators for providing fuel and make up gas to the FID at pressures and flow rates as specified in Table 1.
      **Table 1.** Pressure and flow specifications of gas supply from gas generators to FID

Gas	Pressure (kPa)	Flow range (mL·min^-1^)
H_2_	420	30–50
Dry air	420	300–600
N_2_	420	10–60
    - Follow Table 2 for a summary of chromatography measurement parameters to be configured for the GC in the “Instrument Setup” page of the Xcaliber software.
      **Table 2.** List of GC measurement parameters

Variable	Unit	Value
Carrier gas	-	He
Inlet mode	-	Split
Injector base temperature	°C	100
Liner	mm	5
Split flow	mL·min^-1^	40
Septum purge	mL·min^-1^	5
Carrier mode	-	Constant pressure
Pressure	kPa	140
Column	-	TG-BOND Q+
Stationary phase	-	Divinyl benzene homopolymer
Column length	m	30
Column diameter	mm	0.32
Column film thickness	μm	10
Oven starting temperature	°C	60
Heating rate	°C·min^-1^	0
Oven final temperature	°C	60
FID base temperature	°C	200
H_2_ flow rate	mL·min^-1^	35
Air flow rate	mL·min^-1^	350
N_2_ flow rate	mL·min^-1^	30
FID range	-	1
FID gain	-	1
Analog filter status	-	On
Sampling depth	mm	25
Syringe filling speed	mL·min^-1^	50
Injection speed	mL·min^-1^	50
Injection depth	mm	50
Needle penetration speed	mm·s^-1^	25
- **Configuration of a processing setup in Thermo Xcaliber software**
  1. Use the “Processing Setup” option to import a “.raw” file for peak identification. The “.raw” file should represent a GC trace from a gas injection consisting of a low concentration C_2_H_4_ gas.
  2. Follow Table 3 for the set of variables to be configured into the software.
    **Table 3.** Parameters for the GC’s software processing in terms of peak identification

Page	Variable	Value
Identification	Detector type	Analog
	Peak detect	ICIS
	Expected time (min)	1.64
	Window (sec)	5.00
	View width	0.75
Detection	Smoothing points	1
	Baseline window	60
	Area noise factor	5
	Peak noise factor	10
	ICIS peak detection	Highest peak
	Minimum peak height (S/N)	3.0
Calibration	Component type	Target compound
	Weighting	Equal
	Calibration curve	Linear
	Units	nL
	Origin	Ignore
	Response	Area
Levels	1	0.075
	2	0.150
	3	0.301
	4	0.752
	5	1.505
	6	3.010
	7	8.809
	8	16.709
	9	24.609
	10	44.359
	11	83.859
  3. An example of a part of the processing setup is depicted in Figure 3 illustrating a retention time of 1.64 min.
    **Figure 3.**
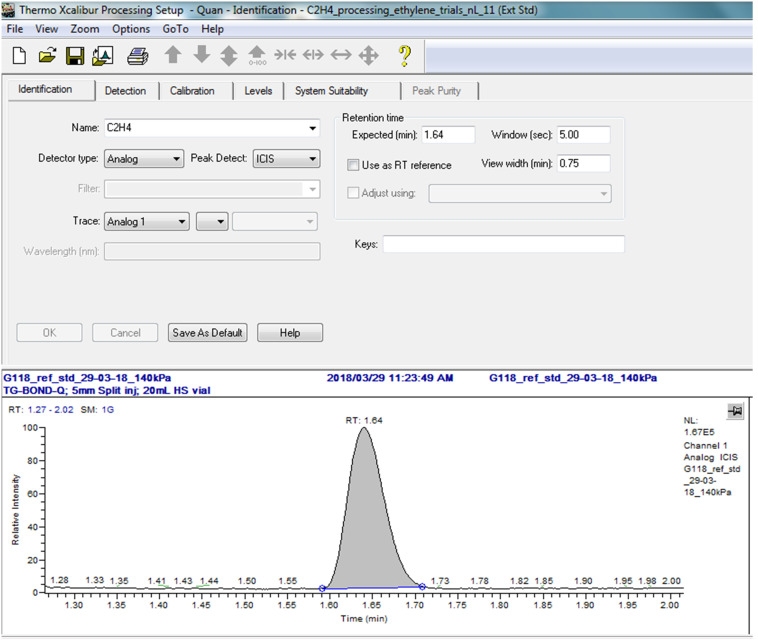
Example of the Xcaliber Processing Setup for peak identification
- **Creation of sequence setups in Xcaliber software for calibration standards and samples**
  1. Create a sequence file using the “Sequence Setup” window in Xcaliber consisting of 11 entries for the calibration standards. It is important to reference the instrument method file under heading “Inst Meth” and the processing method under “Proc Meth”.
  2. Give a file path and name for the calibration file in the sequence table under the heading “Cal File”.
  3. Create a sequence file for the planned number of samples and enter the name of the calibration file to be used under the heading “Cal File”.
  4. Note that the tray holder and slot position is only important for calibration standards 1 to 6 since the remainder of injections are done manually.
  5. Details to create sequence files are further illustrated in Figure 4.
    **Figure 4.**
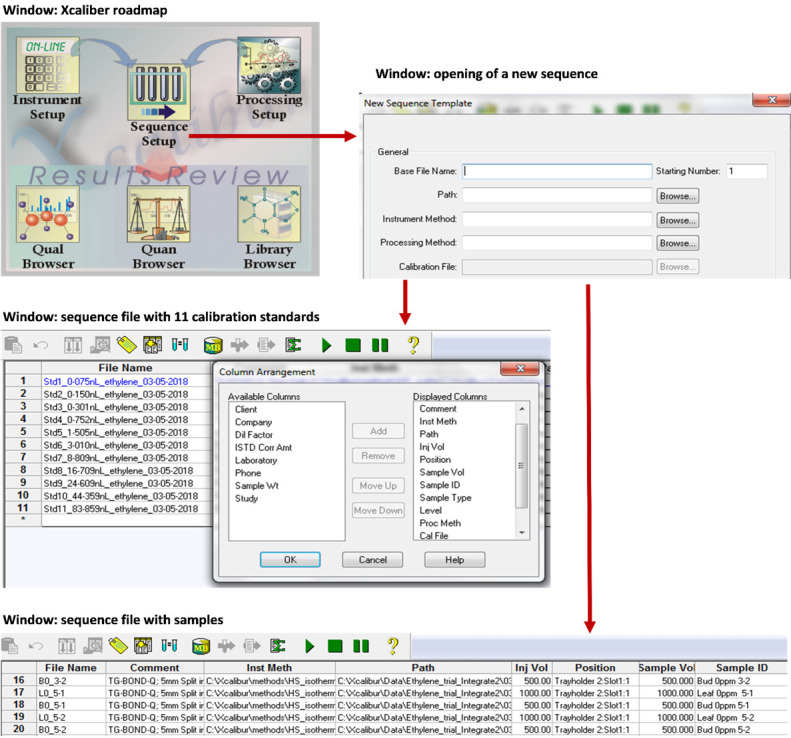
Steps to follow in the Xcaliber software to create sequence files
- **Collection of plant material**
  1. Detach two leaflets (one leaflet each from two terminal shoots) from the compound leaf closest to the terminal bud by manually breaking them off at the natural abscission layer. Remove up to 20 terminal buds (4–8 mm in length, depending on bud stage and size) with straight sharp Iris scissors. Insert the leaflets and buds into the 20 and 2 mL vials, respectively, as illustrated in Figure 5. This will yield minimum masses for buds and leaves of 0.2 and 1.1 g, respectively. The actual mass of plant material is not critical because the calculated rate of C_2_H_4 _release is normalized to unit mass of plant material. Refer to equation 5.
    **Figure 5.**
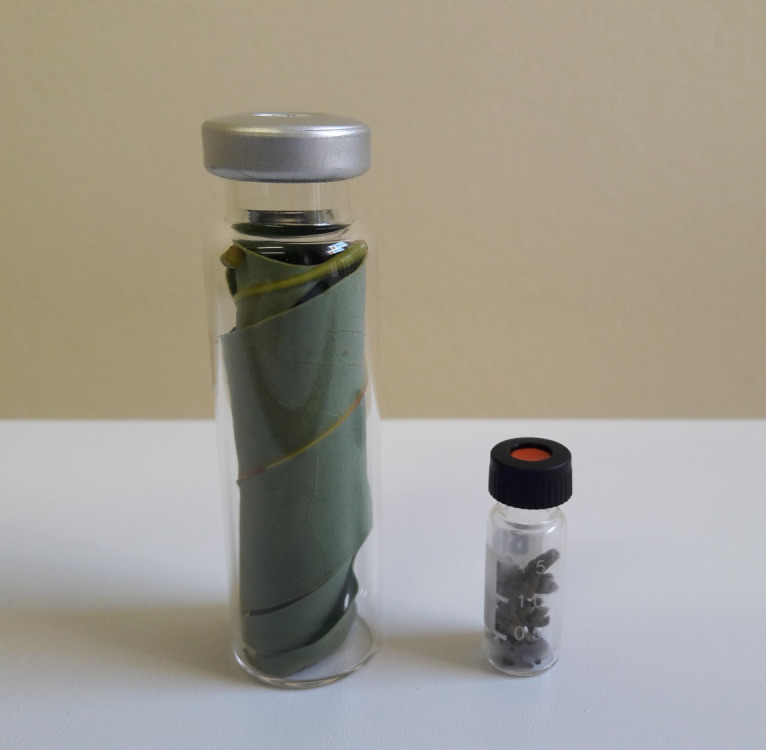
Leaves and buds sealed in 20 mL (left) and 2 mL (right) vials, respectively
  2. Document the time of harvesting of each individual sample as reference for the standardization of the 10 min duration required for the release of wound ethylene (see next step).
  3. Leave vials open for 10.0 ± 0.5 min after insertion to allow for release of wound ethylene. This can readily be achieved at the location of harvesting under ambient conditions.
  4. After the 10-min equilibration time, seal the 20 mL vials with 20 mm Si/PTFE septa and 20 mm aluminum crimp caps using the 20 mm hand crimper.
  5. Ensure that the septa inside the crimp caps have the PTFE face downwards as illustrated in Figure 6, i.e., the PTFE face will contact the beveled edge of the headspace vial.
    **Figure 6.**
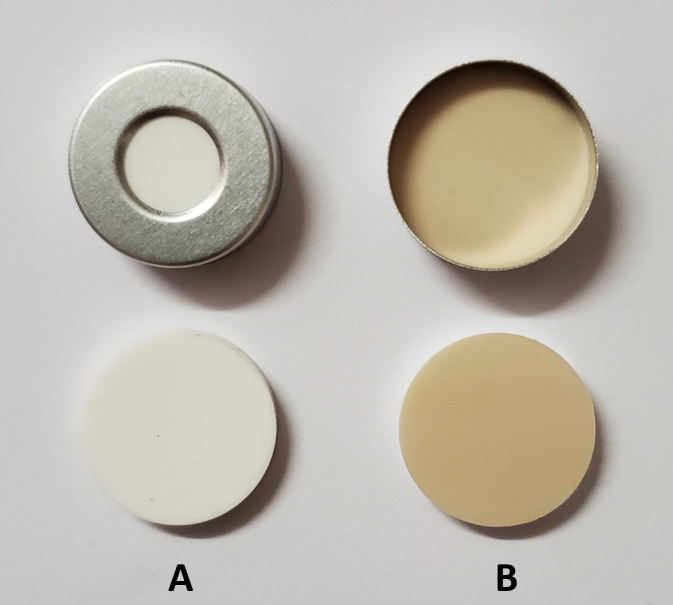
Position of a Si/PTFE septum inside a crimp cap. A. Top view. B. Bottom view.
  6. Close the screw caps on the 2 mL vials hand-tight following the 10-min equilibration time.
  7. Document the time when each individual vial is sealed as starting time of the 3-h incubation period (see step H1).
- **Preparation and GC measurements of C_2_H_4_ calibration standards by headspace vial dilution**
  1. For calibration standards 1 to 6, seal 6 × 20 mL headspace vials containing ambient air with 20 mm magnetic crimp caps and 20 mm Si/PTFE septa.
  2. With a 2-stage regulator connected to a 79 μL·L^-1^ C_2_H_4_ gas cylinder, seal the low-pressure outlet with a custom sized Si/PTFE septum inside a nut.
  3. Set the outlet pressure of the regulator to 50 kPa. It is important not to exceed 100 kPa as it presents a danger to equipment and personnel due to the nature of the configuration of the outlet. The initial pressure setting is done as follows:
    - The cylinder shut-off valve is in the closed position.
    - Turn the control knob on the regulator fully counterclockwise to have the regulator outlet in the closed position.
    - Open the cylinder shut-off valve and close it immediately again; this will charge the inlet stem and regulator with the cylinder gas.
    - The reading on the regulator’s high-pressure gauge now indicates the cylinder pressure.
    - Turn the regulator’s control knob slowly clockwise until the low-pressure gauge reads 50 kPa.
  4. Purge the regulator three times with the analytical gas from the valve side to the low-pressure outlet of the regulator as follows:
    - It is important to have the cylinder shut-off valve closed at the start of the procedure.
    - Insert a 23 gauge needle through the septum on the low-pressure outlet.
    - Monitor the pressure on the high-pressure gauge of the regulator until it decreases close to the zero reading. Remove the needle just prior to the gauge reaching zero. This implies that the needle should be removed before the low-pressure gauge decreases from 50 kPa. A small decrease in outlet pressure from the 50 kPa setting can be tolerated as long as the gauge pressure remains positive to prevent air from flowing back into the outlet of the regulator.
    - Open the cylinder valve and close it again to charge the inlet stem and regulator once more.
    - Insert the needle through the septum again and repeat steps 4a–4d three times. The necessity of purging in triplicate is to ensure that all air inside the inlet stem and regulator is replaced by gas from the cylinder and thereby ensuring that the actual analytical gas is aspirated when preparing standards.
  5. Use the 100 μL and 2,500 μL syringes to aspirate volumes of analytical gas from the low-pressure outlet of the regulator that is connected to the 79 μL·L^-1^ C_2_H_4_ gas cylinder. Figure 7 presents the technique for extracting gas from a cylinder using a gastight syringe.
    **Figure 7.**
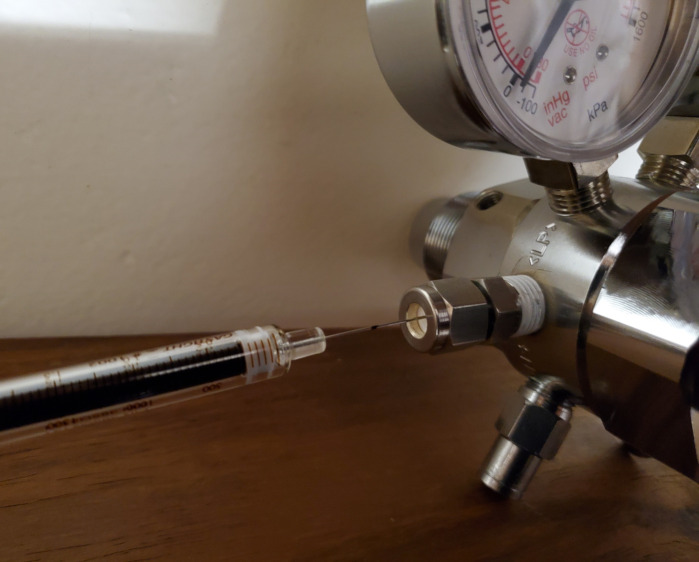
Aspirating a volume of C_2_H_4_ through a septum from a pressure regulator
  6. The required volumes, V_syringe_, for injection into the 20 mL headspace vials are specified in column 3 of Table 4, with a photographic illustration of the use of a 100 μL syringe given in Figure 8. A needle insertion depth of 30 mm is sufficient.
    **Figure 8.**
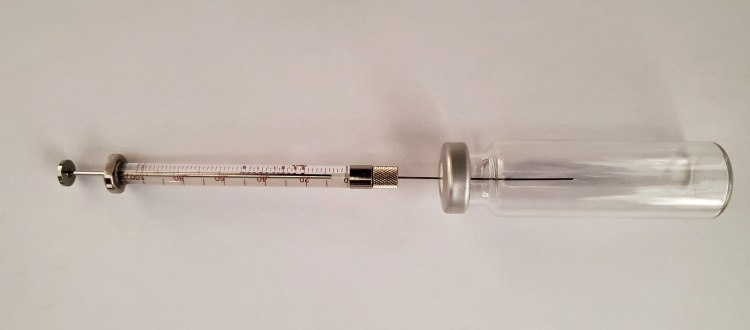
Use of a 100 μL syringe to dilute C_2_H_4_ gas in a headspace vial
  7. The syringe technique should consist of a volume extraction that exceeds the specified volume. The syringe plunger can then be pushed back to the required volume to eliminate pressure effects inside the syringe.
  8. Inject 1 mL volumes of standards 1 to 6, V_inj_, into the GC using the Triplus autosampler with the configurations as described for the instrument method, as well as processing and sequence setups. An automated injection step using a headspace tool is presented by the photograph in Figure 9.
    **Figure 9.**
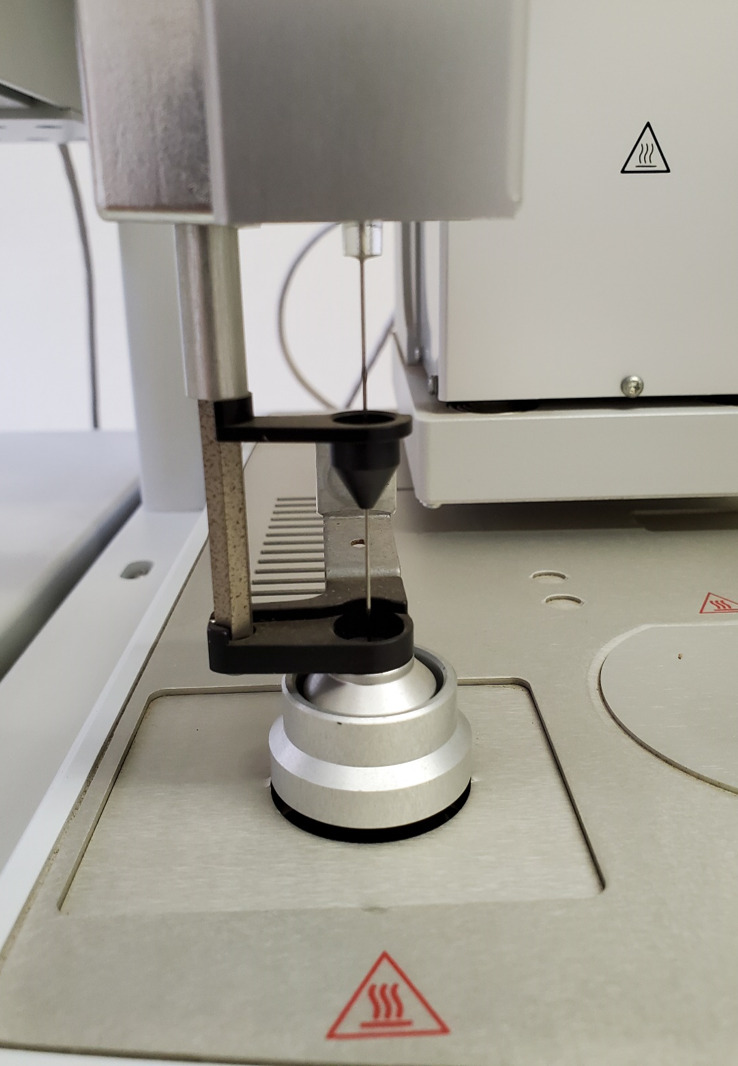
Automated injection with a headspace tool
  9. Since an FID is a mass sensitive detector, the calibration is performed based on the quantity of analytical gas, i.e., C_2_H_4_, injected into the split liner, V’_inj_. The values presented in Table 4 can be obtained by means of equations 1 to 3:

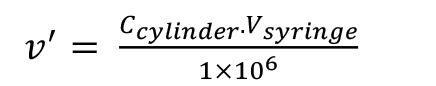

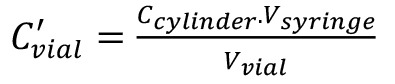

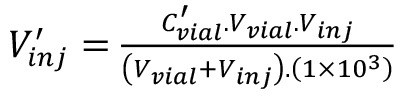

    Definition of the variables are as follows:
    C_cylinder _= concentration of C_2_H_4_ in the gas cylinder (μL·L^-1^),
    V_vial_ = volume of headspace vial (mL),
    V_syringe_ = volume aspirated into syringe (μL),
    v’ = volume C_2_H_4_ in the syringe (μL),
    C’_vial_ = concentration C_2_H_4_ in headspace vial (nL·L^-1^),
    V_inj_ = total volume gas injected into the GC liner (mL), and
    V’_inj_ = volume of C_2_H_4_ injected into the GC liner (nL).
    **Table 4.** Required vial and syringe volumes to prepare standards 1 to 6 in headspace vials

C_cylinder_ (μL·L^-1^)	V_vial_ (mL)	V_syringe_ (μL)	v’ (μL)	C’_vial_ (nL·L^-1^)	V_inj_ (mL)	V’_inj_ (nL)
79	20	20	1.580 × 10^-3^	79	1	0.075
79	20	40	3.160 × 10^-3^	158	1	0.150
79	20	80	6.320 × 10^-3^	316	1	0.301
79	20	200	1.580 × 10^-2^	790	1	0.752
79	20	400	3.160 × 10^-2^	1,580	1	1.505
79	20	800	6.320 × 10^-2^	3,160	1	3.010
- **Preparation and GC measurements of C_2_H_4_ calibration standards by in-situ syringe dilution**
  1. Standards 7 to 11 are prepared by means of in-situ dilution in a 2,500 μL gastight syringe.
  2. Aspirate volumes of gas, V_syringe_, from the cylinder as indicated in column 1 of Table 5. Apply again the technique of extracting volumes in excess of the required and then moving the plunger to the required volume.
    **Table 5.** Required volumes to prepare standards 7 to 11 using in situ syringe dilution

V_syringe_ (μL)	V_dead_ (μL)	C_cylinder_ (μL·L^-1^)	V’_inj-IS_ (nL)
50	61.5	79	8.809
150	61.5	79	16.709
250	61.5	79	24.609
500	61.5	79	44.359
1000	61.5	79	83.859
  3. Thereafter “dilute” the analytical gas with air by setting the plunger to the 1000 μL mark.
  4. Inject 1 mL of standards 7 to 11 manually into the GC during a programmed calibration sequence in the Xcaliber software.
  5. Limit the insertion depth of the needle into the split liner to 50 mm.
  6. Observe that a dead volume, V_dead_, of the 2,500 μL syringe that contributes to the volume of analytical gas is indicated in Table 5. This value is syringe-specific.
  7. Calculate the volume of C_2_H_4_, V’_inj-IS_, injected into the liner in nL units by means of equation 4:

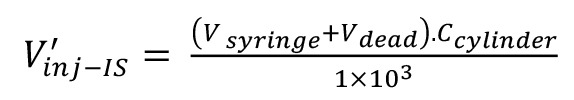

- **Preparation and GC measurements of blanks and C_2_H_4_ quality control (QC) standards**
  1. For the 20 mL headspace QC vials, seal 4 × 20 mL headspace vials, containing only ambient air, with 20 mm aluminum crimp caps and 20 mm Si/PTFE septa.
  2. For the 2 mL screw top QC vials, close the screw tops of 4 × 2 mL vials. These vials also only contain ambient air.
  3. For the C_2_H_4_ QC standards, use a 100 μL gastight syringe to aspirate 20 μL and 80 μL of the C_2_H_4_ standard to prepare three technical replicates of the 2 mL and 20 mL vials, respectively.
  4. To sample the respective gas volumes for QC samples, aspirate from the regulator in excess of the required volume, move the plunger back to the exact required volume and then inject into the respective vials.
  5. Refer to Table 6 for data on the prepared C_2_H_4_ QC samples.
    **Table 6.** Required vial and syringe volumes to prepare C_2_H_4_ QC samples

C_cylinder_ (μL·L^-1^)	V_vial_ (mL)	V_syringe_ (μL)	v’ (μL)	C’_vial_ (nL·L^-1^)	V_inj_ (mL)	V’_inj_ (nL)
79	2	20	1.580 × 10^-3^	790	0.5	0.316
79	20	80	6.320 × 10^-3^	316	1	0.301
  6. The total number of prepared QC vials consists of 1 × 2 mL blank (containing ambient air), 1 × 20 mL blank (containing ambient air), a 3 × 2 mL C_2_H_4_ QC standards (containing 20 μL of C_2_H_4 _standard), and a 3 × 20 mL C_2_H_4_ QC standards (containing 80 μL of C_2_H_4 _standard).
  7. Perform gas sampling from the QC vials using disposable 3 mL syringes with luer valves, each connected to a 23 gauge needle of 51 mm length. An example of a syringe with a luer valve can be seen in Figure 10.
    **Figure 10.**
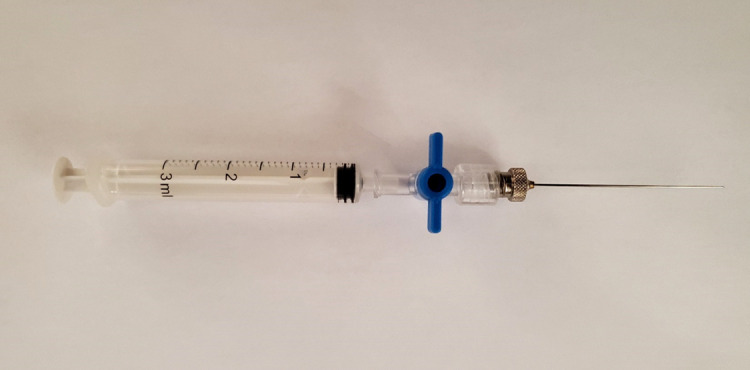
Disposable 3 mL syringe with luer lock and attached needle
  8. Aspirate 1 mL and 0.5 mL from the 20 mL and 2 mL vials, respectively.
  9. An advisable syringe technique is to cycle the required volume two times with the needle stationary through the septum prior to aspirating the required volume.
  10. Take care to close the luer valve while holding the plunger in position for the required aspiration volume. This is specifically relevant when sampling 0.5 mL from a 2 mL vial, i.e., air pressure will restore the plunger to a volume less than extracted, which is prevented by ensuring that the valve is closed prior to releasing the syringe plunger. The technique to achieve this is demonstrated by the sequence of photographs in Figure 11.
    **Figure 11.**
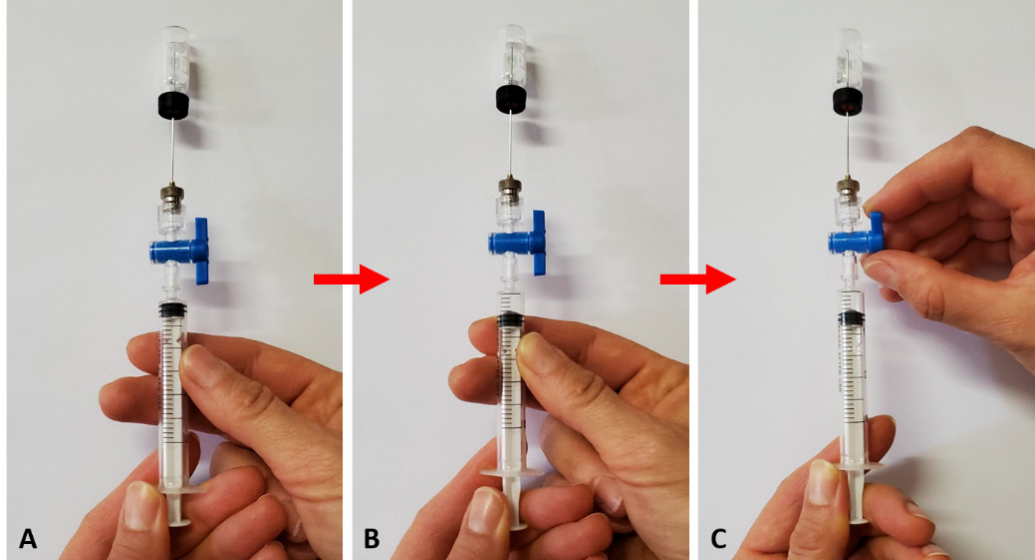
Technique to aspirate a gas sample from a small volume (2 mL) vial with a disposable syringe. A. Positioning the needle through the septum with the luer lock open. B. Withdrawing of a gas sample by moving the plunger outwards. C. Closing the valve’s luer lock while holding the plunger in position at the required volume.
  11. Use a clean 3 mL syringe to flush needles that are alternating between vials to ensure any C_2_H_4_ gas is expelled from the needles.
  12. Inject the QC samples manually into the GC liner during a programmed sequence.
  13. The syringe can be inserted with its full length through the injector’s septum, i.e., 51 mm, to agree with the programmed injection depth setting of 50 mm. The manual injection process is illustrated by the sequence of photographs in Figure 12.
    **Figure 12.**
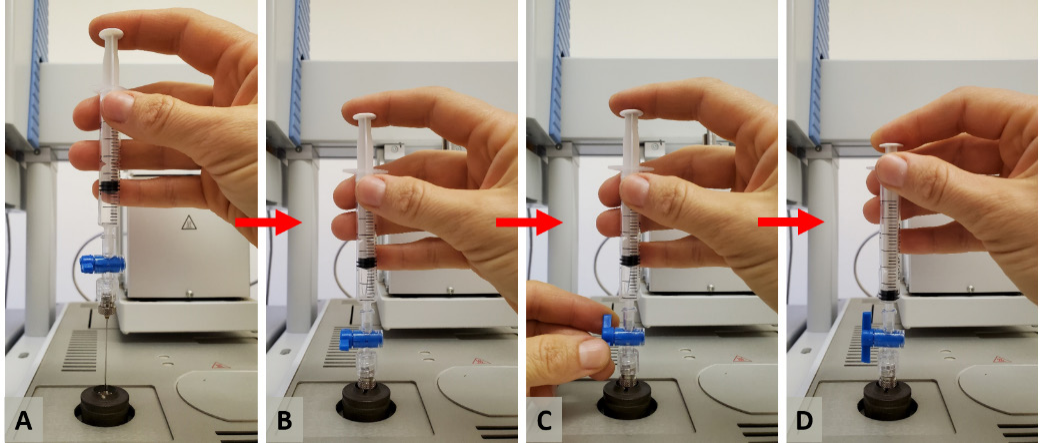
Sequence of steps for manual injection. A. Positioning the needle on top of the injector. B. Inserting the needle through the septum. C. Opening the valve’s luer lock while maintaining control of the syringe’s plunger to prevent the head pressure from pushing the plunger upwards. D. Pressing the plunger down to inject the gas sample into the injector.
- **GC measurements of C_2_H_4_ evolved from leaves and buds**
  1. Perform gas sampling 3.00 ± 0.05 h after sealing vials using disposable 3 mL syringes with luer valves connected to 23 gauge needles of 51 mm length. The 3-h incubation period for each vial consists of the total time the plant material is allowed to release C_2_H_4_ under sealed conditions. It includes time between sealing of the vials at the location of collection (orchard), transport to the laboratory, and monitored time on the laboratory bench pending gas sampling. The 3-h incubation period is effectively terminated once gas is sampled from a vial using a syringe with a luer valve.
  2. An advisable syringe technique is to cycle the required volume twice with the needle stationary through the septum prior to aspirating the final required volume.
  3. The volumes to aspirate are 1 mL and 0.5 mL from the 20 mL and 2 mL vials, respectively. Piercing of the plant material should not occur. In the case of leaves, this is prevented by the technique with which the leaves are inserted into the vial, i.e., predominantly against the wall of the vial. In the case of the buds, the insertion of the needle is visibly controlled to avoid contact with the plant material. Moreover, the needle has a dome tip with a side hole.
  4. Take care to close the luer valve while holding the plunger in position for the required aspiration volume as described under G10.
  5. Document the sampling time of each vial.
  6. Use a clean 3 mL syringe to flush needles that are alternating between sample vials to ensure any C_2_H_4_ gas is expelled from the needles.
  7. Once a headspace volume is aspirated into a syringe and locked, the syringe can be stored for the duration of the sequence of chromatography measurements.
  8. Inject gas samples manually into the GC in accordance with a programmed sequence file.
  9. The needle can be inserted with its full length through the injector’s septum as described under G13.
- **Weighing of plant material**
  1. Following chromatography analysis, remove the 20 mL vial caps with a pair of pliers and unscrew the caps of the 2 mL vials for weighing the vials individually with their contents.
  2. Remove the contents and weigh the masses of the empty 20 mL and 2 mL vials as associated with each sample number.

## Data analysis

1. Use the “Quan Browser” function in the Xcaliber software to retrieve quantitative reports of calibration standards and samples as exemplified in Figures 13 and 14. Note from Figure 14 that the peak status of the “blank” is indicated as “not found” by the software. This confirms that C_2_H_4_ is absent, hence no retention time can be identified, or peak area calculated.
  **Figure 13.**
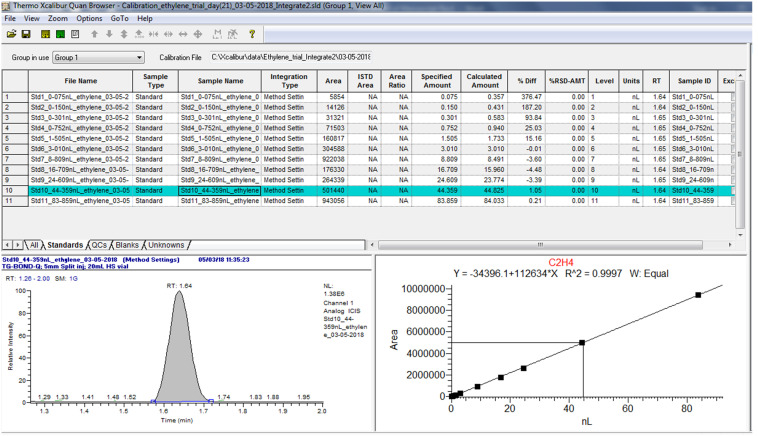
Example of quantitative data of calibration standards from the Xcaliber “Quan Browser” software
  **Figure 14.**
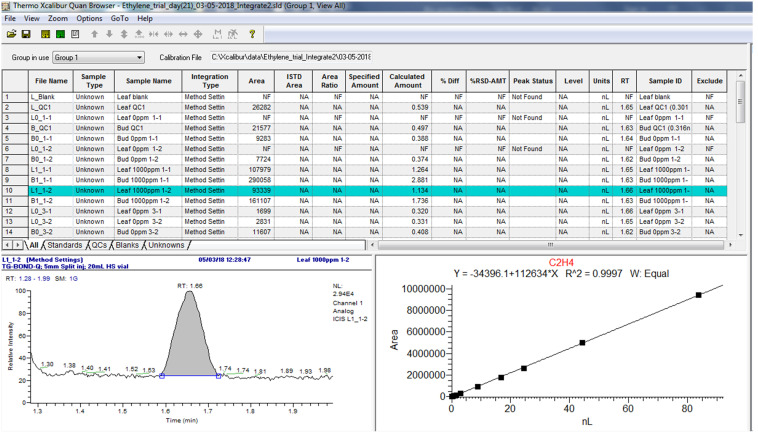
Example of quantitative data of samples from the Xcaliber “Quan Browser” software
2. In the “Quan Browser” window, use the file menu “Export data to Excel” to create an Excel document with the measured data for individual sequences.
3. Perform data processing in Excel to calculate the specific C_2_H_4_ production rate, R, in μL·kg^-1^·h^-1^, using equation 5. Assignment of variables is as follows:
  GC_C2H4_ = GC analyzed C_2_H_4_ volume, i.e., calculated amount in Quan Browser (nL),
  V_vial_ = volume of vial (mL),
  V_inj_ = total volume of gas injected with syringe (mL),
  m_vc_ = mass of vial and contents (g),
  m_v_ = mass of empty vial (g) and
  t = difference between time of sealing vial and, t_s_, time of gas aspirated from vial (h), t_a_.

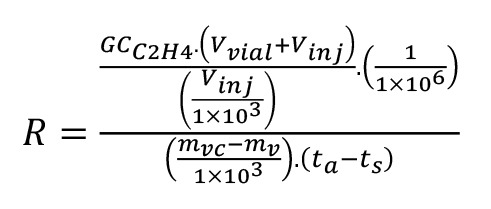

## Notes

1. Depending on the size of the plant material to be analyzed, the number of plant organs and/or vial sizes can be adjusted.
2. A minimum of five biological replicates, with sub-samples per replicate, is advisable as considerable variation in C_2_H_4 _readings can be expected after exogenous C_2_H_4 _application, e.g., through ethephon application.
3. The duration for wound ethylene release and incubation period can differ when plant tissues of other crops and vial volumes, respectively, are used as study material. It is advisable to determine these periods before the experiment is conducted.
4. As a cautionary point, it is important to perform timing accurately to ensure that the incubation time is the same for all samples.
5. It is advisable to perform ad-hoc qualitative chromatography of typical samples prior to systematic quantitative measurements to assess the expected maximum and minimum levels of C_2_H_4_. The observed peak areas can serve as a guide to the required calibration ranges.
6. Table 3 lists the retention time of C_2_H_4_ as 1.64 min. Slight deviation from the expected 1.64 min may still occur even if the protocol is reproduced in its totality. The retention time for C_2_H_4_ (or any compound of interest) can be identified or confirmed by an increase in peak area following the measurement of GC traces for a sequence of standards with increasing C_2_H_4_ concentration.
7. Calibration curves with a correlation coefficient >99.0% can be easily attained with the described technique.
8. It is worthwhile to include blank and QC injections intermittently during a sequence to calculate precision data and to validate the measurements of unknowns.
